# Reduced harm aversion relates to antisocial behaviors and orbitofrontal atrophy in dementia patients

**DOI:** 10.1002/alz.70623

**Published:** 2025-09-08

**Authors:** Tony X. Phan, Jayden J. Lee, Jenifer Z. Siegel, Hongbo Yu, Jerica E. Reeder, Simon Vandekar, Ciaran M. Considine, David H. Zald, R. Ryan Darby

**Affiliations:** ^1^ Department of Neurology Vanderbilt University Medical Center Nashville Tennessee USA; ^2^ Department of Psychology Jerome L. Greene Science Center Columbia University New York New York USA; ^3^ Department of Psychological and Brain Sciences Psychology Building University of California Santa Barbara California USA; ^4^ Department of Biostatistics Vanderbilt University Medical Center Nashville Tennessee USA; ^5^ Center for Advanced Human Brain Imaging Research Rutgers University Piscataway New Jersey USA

**Keywords:** antisocial behavior, behavioral variant frontotemporal dementia, empathy, harm aversion, social cognition

## Abstract

**INTRODUCTION:**

Antisocial behaviors occur in dementia, but the underlying neurocognitive mechanisms remain underexplored. We administered a decision‐making task measuring patients’ harm aversion by offering options to shock themselves or another person in exchange for money, hypothesizing that task performance would relate to antisocial behaviors and ventromedial/orbitofrontal cortex (vmPFC/OFC) atrophy.

**METHODS:**

Among 43 dementia patients (*n* = 23 behavioral variant frontotemporal dementia [bvFTD], *n* = 20 Alzheimer's disease [AD]), we used linear regressions to measure relationships between harm aversion and antisocial behavior, psychopathic personality traits, socioemotional functions, and vmPFC/OFC cortical thickness, controlling for age, sex, and cognitive dysfunction.

**RESULTS:**

BvFTD patients demonstrated reduced aversion to harming others and themselves versus AD patients. Reduced aversion to harming others was associated with non‐aggressive antisocial behaviors, psychopathic personality traits, impaired empathic concern, impaired perspective taking, and right vmPFC/OFC atrophy.

**DISCUSSION:**

Changes to harm aversion are associated with right frontopolar atrophy and rule‐breaking criminal behavior in dementia patients.

**Highlights:**

- Patients with behavioral variant frontotemporal dementia demonstrate reduced aversion to harming others compared to patients with AD.
- Reduced aversion to harming others was associated with non‐aggressive behavioral changes, psychopathic personality traits, impaired empathic concern, and impaired perspective taking.
- Reduced aversion to harming others was associated with atrophy in the right vmPFC and OFC, specifically in medial Brodmann area 10.

## 1 BACKGROUND

Patients with dementia can develop changes to decision‐making, judgment, and social behavior,^1^, ^2^, ^3^ resulting in antisocial behaviors in severe cases. Antisocial behavior is particularly common in behavioral variant frontotemporal dementia (bvFTD), with estimated rates ranging from 37% to 91%.^4^, ^5^, ^6^, ^7^, ^8^, ^9^ Antisocial behaviors in dementia can have a profound impact on patients, family members, and society and contribute to increased caregiver burden,^10^ functionality,^11^ and loss of personhood and identity.^12^ Despite this, our understanding of the decision‐making and neural mechanisms leading to antisocial behaviors in patients with dementia remains incomplete. Understanding the mechanisms leading to “acquired” antisocial behavior in neurological patients may also lend insight into how criminality develops in persons without clear neuropathology, a problem that is estimated to cost society upwards of $1 trillion annually.^13^

In addition to executive dysfunction and impaired social cognition,^14^, ^15^, ^16^ bvFTD patients also demonstrate differences on moral decision‐making tasks involving decisions regarding whether to cause harm to another person, and on neuroeconomic, value‐based decision‐making tasks involving decisions regarding monetary gains and losses.^17^, ^18^, ^19^, ^20^, ^21^, ^22^ This prior work suggests bvFTD patients may have less aversion to negative outcomes in general, and more specifically less aversion to harming other persons in social contexts. Abnormal performance on moral and neuroeconomic decision‐making tasks has been linked to antisocial behavior in other populations, including patients with ventromedial prefrontal cortex/orbitofrontal cortex (vmPFC/OFC) brain lesions^23^ and persons with psychopathic personality disorder.^24^, ^25^ However, the relevance of these decision‐making processes to the development of abnormal social behaviors in patients with dementia remains unknown.

Previously, Crockett et al. designed a task that integrates moral dilemmas within a neuroeconomics framework.^26^, ^27^, ^28^ The task asks subjects to choose whether to deliver more shocks to a stranger (or oneself) in exchange for more money or fewer shocks in exchange for less money. By varying the difference in shocks and monetary gain across trials, this task provides a quantitative measure of a subject's relative aversion to causing harm to another person (or oneself). Here, we use this paradigm to determine whether harm aversion during a decision‐making task relates to antisocial behavior, psychopathic personality traits, and/or socioemotional dysfunction in patients with dementia. Further, given prior work demonstrating an association between the vmPFC/OFC and both moral and value‐based decision‐making, we tested whether harm aversion would relate to brain atrophy within the vmPFC/OFC in patients with dementia.

## 2 METHODS

### 2.1 Patient selection

Patients for this study were recruited from the Vanderbilt University Medical Center Department of Neurology's Behavioral and Cognitive Neurology clinic. (See Supplementary Methods for further details.)

We chose to include both patients with bvFTD and AD to increase the variance across task performance, social behavior, and locations of neurodegeneration. A disease comparison group offers advantages over a healthy control group because one can test whether task performance is linked specifically to social decision‐making impairments in bvFTD, versus whether task performance relates more generally to cognitive dysfunction that can be seen in other types of dementia like AD.

### 2.2 Harm aversion task

**FIGURE 1 alz70623-fig-0001:**
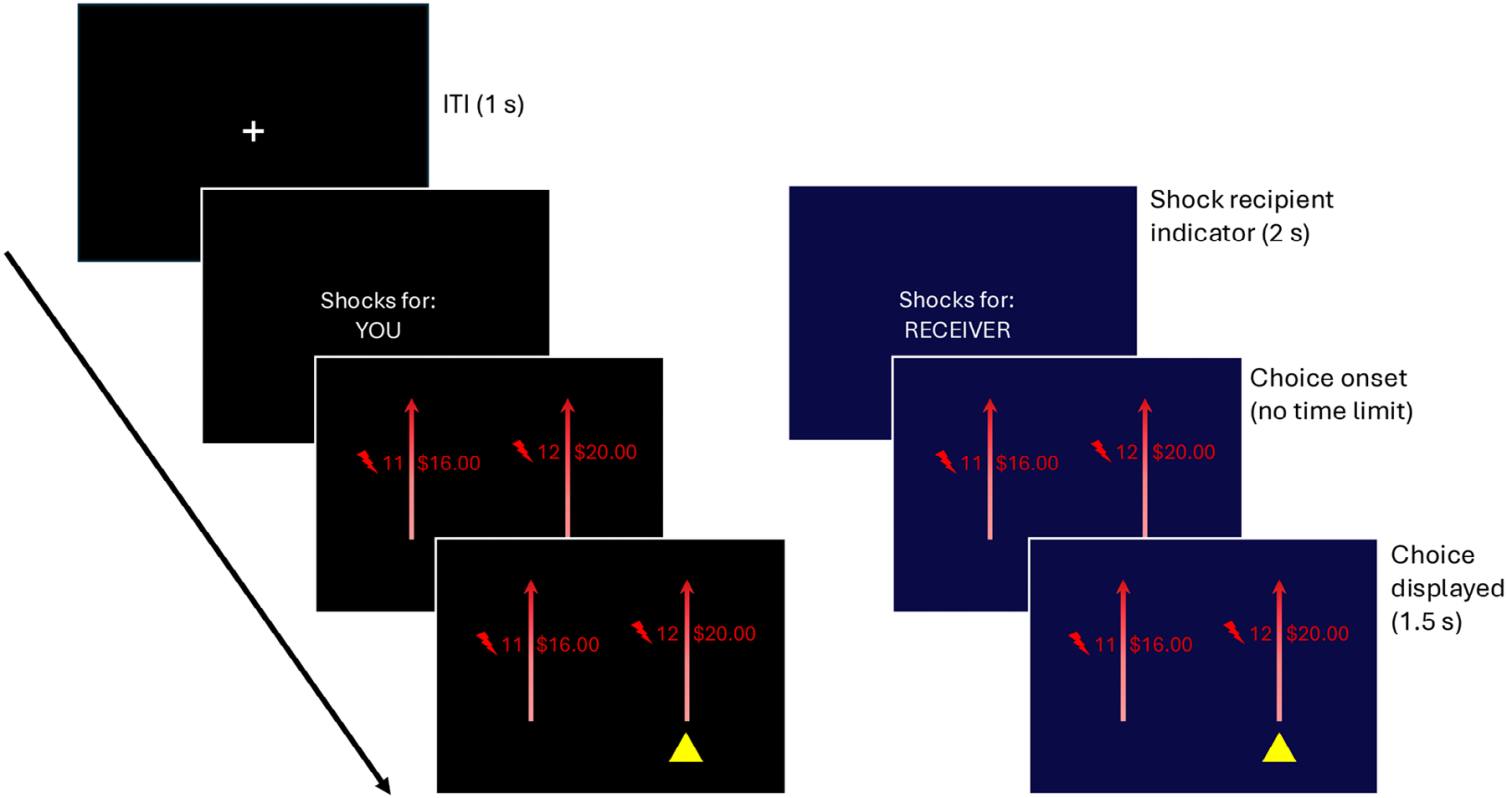
Summary of harm aversion task. A task was used to measure subjects’ aversion to harming themselves or others. For each trial, the participant is informed whether the recipient of the shocks will be the participant (“you”) or another person (“receiver”). The participant is then asked to choose between two options with a corresponding number of shocks to the participant/receiver and money to the participant.

This computer‐based task is modified from Crockett et al.^27^ (Figure 1). On each trial, subjects are asked whether to deliver a higher number of shocks in exchange for a higher amount of money or a lower number of shocks at the expense of a smaller amount of money.  The recipient of the shock (self vs other) was counterbalanced across 82 trials; however, the subject was always the recipient of the money. In the original experiments, subjects and an unknown stranger were administered shocks at the end of the experiment to make the decisions more salient. However, to minimize the risk to our patients, no shocks were delivered as part of the current study, and no deception was used to make the subjects believe that shocks would be delivered.

Computational modeling was used to estimate each subject's harm aversion to oneself (*κ*_self_) versus others (*κ*_other_) using the following equation:

ΔV=(1−κ)Δm−κΔs

where Δ*V* is the difference in subjective value, and Δ*m* and Δ*s* are the differences in money and shocks between the two options.^26^, ^27^

### 2.3 Antisocial behavior measure

The Social Behavior Questionnaire (SBQ) is an informant‐based measure that assesses 26 antisocial behaviors common in bvFTD patients, including lying, stealing, aggression, and inappropriate sexual behaviors.^8^ For each item, behaviors are rated on a Likert scale from 0 (absent) to 5 (severe). Based on our prior study, the SBQ can be divided into distinct factors associated with non‐aggressive (seven items) and aggressive (10 items) antisocial behaviors,^8^ which are associated with different deficits in social cognition.^29^

### 2.4 Psychopathic personality traits measure

The Levenson Self‐Report Psychopathy (LSRP) scale was used to assess patients’ levels of psychopathy.^30^ The scale was administered to study informants instead of patients due to concerns that patients would be unaware of the extent and severity of their impairments. It consists of 26 items, of which seven are reverse scored. As was previously done, instead of the standard 4‐point scale, items were rated on a 5‐point Likert scale (1 = strongly disagree, 2 = somewhat disagree, 3 = neutral, 4 = somewhat agree, 5 = strongly agree), which was more comparable to the scale used for SBQ item severity.^31^, ^32^

### 2.5 Socioemotional measures

The Interpersonal Reactivity Index (IRI) is a questionnaire where 28 items are rated on a Likert scale from 1 (does not describe well) to 5 (describes very well).^33^ Originally developed as a patient questionnaire, the measure has been adapted and extensively used as an informant‐based measure in patients with dementia due to concerns of patients being unaware of their deficits.^34^, ^35^, ^36^, ^37^ Consistent with prior studies in bvFTD, we focused on IRI scores for empathic concern and perspective taking.^38^

**RESEARCH IN CONTEXT**

1. **Systematic review**: The authors reviewed literature on frontotemporal dementia, antisocial behavior, and task‐based methods to assess harm aversion and antisociality. Although prior studies indicated dementia could cause behavioral issues and evaluated the use of task‐based methods for harm aversion in cognitively normal individuals, the relationship between harm aversion and antisocial behavior in dementia patients remains poorly understood.
2. **Interpretation**: Our findings suggest neurological and cognitive mechanisms for the development of antisocial behavior in dementia patients. Results are consistent with prior studies relating vmPFC/OFC and social decision‐making. Additionally, these results further refine the neuropsychiatric understanding and neuroanatomical localization of antisocial behavior.
3. **Future directions**: Our manuscript suggests potential new directions for studies to clarify understanding of behavioral changes in dementia. These include (a) task‐based measurements of social decision‐making in other dementia subtypes and (b) investigating aversion to negative stimuli in social versus non‐social settings for bvFTD.

### 2.6 Other assessments

The Montreal Cognitive assessment (MoCA) was administered[Fig alz70623-fig-0001] to each patient as a generalized measure of cognitive function.^39^ Prior research demonstrated adequate correlations between MoCA subtask performance and standard neuropsychological measures of corresponding cognitive constructs,^40^ supporting the use of MoCA subtask items for examining specific cognitive domains. To assess frontal‐mediated cognitive functioning, we created an Attention–Concentration–Working Memory–Executive Functioning (ACWMEF) construct score from MoCA subtask items, including Attention–Concentration–Working Memory subtasks (Digits Forward, Digits Backward, Sustained Attention, Subtraction) and Executive Functioning subtasks (Trail Making Test Part B [TMT‐B] adaptation, Phonemic Fluency, Abstraction), based on factor analytic findings from a large‐scale study of healthy controls and mild cognitive impairment (MCI)/AD patients (*N* = 830).^41^

### 2.7 Magnetic resonance imaging (MRI) imaging and analysis

Structural T1w MRI scans were acquired on Philips Achieva 3.0T scanners using the three‐dimensional (3D) T1 MPRAGE protocol with the shortest repetition time (TR) and shortest echo time (TE), flip angle = 8°, and 1 × 1 × 1 mm^3^ voxel size. One MRI scan was acquired at the shortest TR, shortest TE, flip angle = 9°, and 1 × 1 × 1 mm^3^ voxel size.

Surface‐based analyses were performed using FreeSurfer version 6.0.0.^42^ T1‐weighted structural MRIs were subjected to recon‐all surface reconstruction to generate estimates of cortical thickness. For each subject and hemisphere, cortical surfaces were registered to fsaverage, a common surface template consisting of ∼164,000 vertices per hemisphere.

### 2.8 Neuroimaging region of interest

We defined a region of interest (ROI) for the vmPFC/OFC using Glasser et al.^43^ multimodal parcellation. (See Supplementary Methods for further details.)

### 2.9 Statistical analysis

Group‐level comparisons of demographic characteristics were performed using ANOVAs and post hoc *t*‐tests for continuous measures, Kruskal–Wallis tests for ordinal measures, and two‐tailed Fisher's exact tests for categorical measures. To assess for group‐level differences in harm aversion between patients with bvFTD and AD, we used linear regression models with harm aversion as the response variable, group (bvFTD vs AD) as the predictor of interest, and age, sex, and cognitive dysfunction (MoCA scores) as covariates of non‐interest. To assess whether harm aversion during the decision‐making task related to other social behavioral measures (antisocial behavior, psychopathic personality traits, socioemotional functions), we used linear regression models with harm aversion as the response variable, each behavioral measure as the predictor of interest and age, sex, and MoCA scores as covariates of non‐interest. For the regressions of harm aversion over the ACWMEF score, MoCA was not included as a covariate of non‐interest to reduce the effect of multicollinearity, given the ACWMEF score is derived from items of the MoCA. Supplementary analyses testing for interactions between condition (self vs other) or group (AD vs bvFTD) and social behavior measures did not find significant interactions, so interaction terms were not included (see Supplementary Methods, Supplementary Results, and Tables S1 to S18 in the Supplementary Materials). Additionally, analyses between harm aversion and social behavioral measures were replicated, grouped by diagnosis (Supplementary Methods).

To assess whether harm aversion related to cortical thickness within the vmPFC/OFC, we used a linear regression model in FreeSurfer with cortical thickness within the vmPFC/OFC ROI as the response variable, harm aversion as the predictor of interest, and age, sex, and MoCA scores as covariates of non‐interest. We additionally performed a vertex‐wise analysis restricted to the right vmPFC/OFC ROI using an equivalent linear model, corrected for multiple comparisons using permutation‐based testing (10,000 permutations, cluster‐forming threshold *p* < 0.05, cluster‐wise significance threshold of *p* < 0.05). We additionally performed a supplementary whole‐brain analysis without restriction to the vmPFC/OFC ROI (10,000 permutations, Bonferroni corrected for number of hemispheres [×2], cluster‐forming threshold *p* < 0.05, cluster‐wise significance threshold of *p* < 0.05).

## 3 RESULTS

### 3.1 Demographics and patient characteristics

Twenty‐three bvFTD and 20 AD patients were included in the current analysis (Table 1). The bvFTD group was younger (61.8 ± 9.1 vs 69.6 ± 8.6 years, *F*[1, 41] = 8.23, *p* = 0.006) had a higher proportion of males (91% vs 50%, Fisher's exact test: *p* = 0.005). There were no differences in MoCA scores between groups (20.8 ± 4.6 vs 19.1 ± 5.0, *F*[1, 41] = 1.45, *p* = 0.23). As expected, patients with bvFTD had higher antisocial behaviors, psychopathic personality traits, and socioemotional dysfunction versus patients with AD (Table 1). Among AD patients, four subjects (20%) had positive SBQ scores, whereas among bvFTD subjects, 21 subjects (91%) had positive SBQ scores, indicating criminal risk behavior.

**TABLE 1:** Demographics table

Variable	bvFTD (*N* = 23)	AD (*N* = 20)	*F* ^a^	*P*	Significant comparisons^b^
Age, mean (SD), years	61.8 (9.1)	69.6 (8.6)	8.23	0.006	AD > bvFTD
Sex, No. (%)				0.005^c^	
Female	2 (9)	10 (50)	—	—	—
Male	21 (91)	10 (50)	—	—	—
Race and ethnicity, No. (%)				0.44^c^	
Black or African American, non‐Hispanic, and non‐Latino	2 (9)	0 (0)	—	—	—
White, Hispanic, or Latino	0 (0)	1 (5)	—	—	—
White, non‐Hispanic, and non‐Latino	20 (87)	17 (85)	—	—	—
White, unknown	1 (4)	2 (10)	—	—	—
Education, No. (%)				0.96^d^	
High school degree or equivalent (e.g., GED)	5 (22)	4 (20)	—	—	—
Some college, no degree	3 (13)	5 (25)	—	—	—
Associate degree (e.g., AA, AS)	3 (13)	0 (0)	—	—	—
Bachelor's degree (e.g., BA, BS)	7 (30)	6 (30)	—	—	—
Advanced degree	5 (22)	5 (25)	—	—	—
Informant relationship, No. (%)				0.44^c^	
Spouse	17 (74)	16 (80)	—	—	—
Child	4 (17)	2 (10)	—	—	—
Other	2 (9)	2 (10)	—	—	—
MoCA, mean (SD)	20.8 (4.6)	19.1 (5.0)	1.45	0.23	—
ACWMEF score, mean (SD)	7.30 (1.6)	6.60 (2.2)	1.43	0.24	—
Total LSRP score, mean (SD)	72.0 (19.)	42.9 (9.3)	38.76	<0.001	bvFTD > AD
LSRP antisocial subscale score, mean (SD)	15.0 (4.3)	10.6 (5.0)	9.27	0.004	bvFTD > AD
LSRP callous subscale score, mean (SD)	10.5 (4.1)	5.05 (1.7)	31.07	<0.001	bvFTD > AD
LSRP egocentric subscale score, mean (SD)	24.6 (9.3)	13.2 (3.1)	27.53	<0.001	bvFTD > AD
IRI perspective‐taking score, mean (SD)^e^	8.27 (4.9)	18.3 (4.9)	33.09	<0.001	AD > bvFTD
IRI empathic‐concern subscale score, mean (SD)^e^	14.5 (4.8)	24.9 (2.8)	57.22	<0.001	AD > bvFTD
Total SBQ score, mean (SD)	15.9 (14.)	2.25 (7.4)	15.64	<0.001	bvFTD > AD
SBQ non‐aggressive subscale score, mean (SD)	6.87 (6.6)	0.400 (1.3)	18.48	<0.001	bvFTD > AD
SBQ aggressive subscale score, mean (SD)	12.0 (10.0)	2.05 (6.9)	13.42	<0.001	bvFTD > AD

Abbreviations: ACWMEF, Attention‐Concentration‐Working Memory‐Executive Functioning; AD, Alzheimer's disease; bvFTD, behavioral variant frontotemporal dementia; LSRP, Levenson Self‐Report Psychopathy scale; IRI, Interpersonal Reactivity Index; MoCA, Montreal Cognitive Assessment; SBQ, Social Behavior Questionnaire.

^a^ Group‐level differences were calculated by ANOVA, df = 1, 41, unless otherwise indicated.

^b^ Significant differences were determined by *post hoc t*‐tests with Holm–Bonferroni correction, unless otherwise indicated.

^c^ Group‐level differences in categorical variables were computed by Fisher test.

^d^ Group‐level differences in ordinal variable measured by Kruskall–Wallis test, *χ* ^2^ = 0.0025, df = 1.

^e^ 15 bvFTD patients, 17 AD patients.

### 3.2 Reduced harm aversion in patients with bvFTD versus AD

**FIGURE 2:**
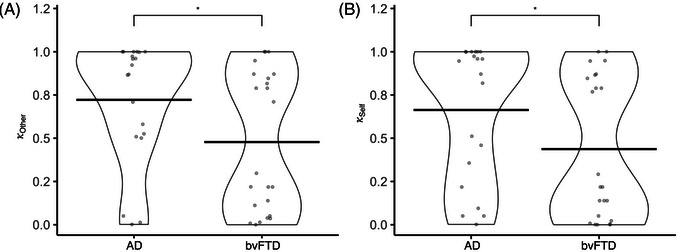
Group‐level differences in harm aversion. A multiple linear model was used to determine whether there was a group‐level difference between AD and bvFTD subjects in their aversion to harming themselves or others. (A) bvFTD subjects were less averse to harming others compared to AD subjects. (B) Likewise, bvFTD subjects were less averse to harming themselves. AD, Alzheimer's disease; bvFTD, behavioral variant frontotemporal dementia.

bvFTD patients showed significantly lower aversion to harming others (*b* = −0.33, SE = 0.13, *t* = −2.60, df = 38, *p* = 0.01, Figure 2A) and to harming themselves (*b* = −0.32, SE = 0.14, *t* = −2.24, df = 38, *p* = 0.03, Figure 2B) during the moral decision‐making task than AD patients.

### 3.3 Reduced aversion to harming others is associated with non‐aggressive but not aggressive antisocial behaviors

**FIGURE 3:**
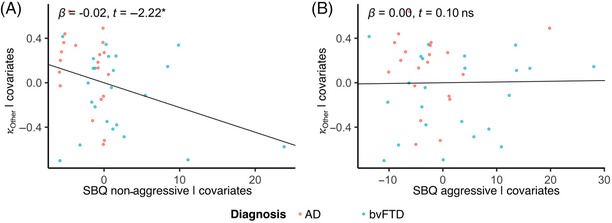
Non‐aggressive behaviors predict reduced aversion to harming others. (A) Non‐aggressive antisocial behaviors in AD and bvFTD patients were associated with reduced aversion to harming others. (B) There was no relationship between aggressive behaviors and aversion to harming others.

Reduced aversion to harming others during the task was significantly associated with non‐aggressive/rule‐breaking behaviors (*b* = −0.022, SE = 0.010, *t* = −2.22, df = 38, *p* = 0.03; Figure 3A), but there was no evidence for an association with aggressive behaviors (*b* = 0.00, SE = 0.01, *t* = 0.10, df = 38, *p* = 0.92; Figure 3B). We did not find evidence that aversion to harming oneself was associated with either non‐aggressive/rule‐breaking behaviors or aggressive behaviors (non‐aggressive: *b* = −0.014, SE = 0.011, *t* = −1.25, df = 38, *p* = 0.22; aggressive: *b* = 0.001, SE = 0.007, *t* = −0.13, df = 38, *p* = 0.90).

### 3.4 Reduced aversion to harming others is associated with psychopathic personality traits

**FIGURE 4:**
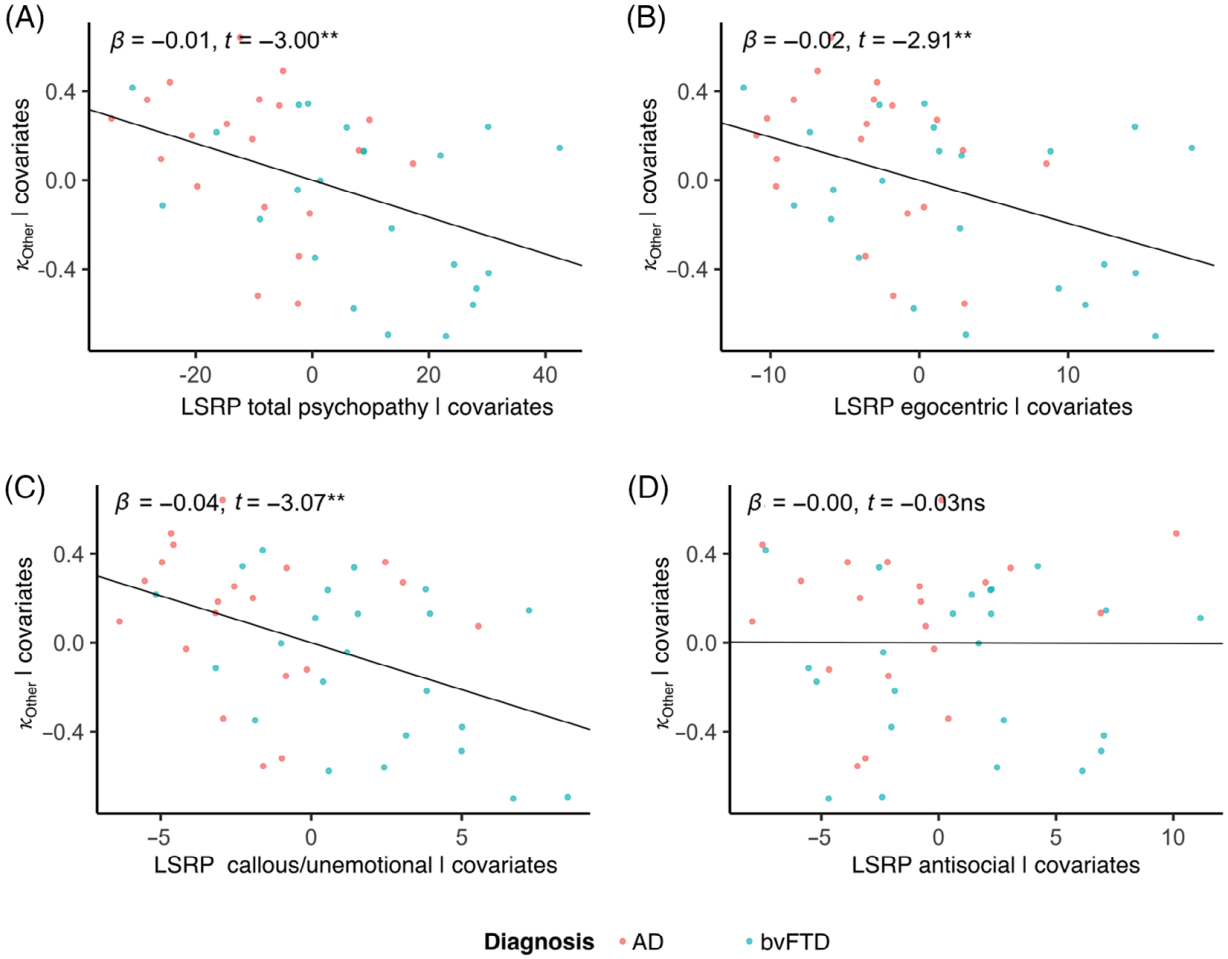
Reduced aversion to harming others is associated with psychopathic personality traits. (A) Among dementia patients, aversion to harming others was associated with (A) total psychopathy and the (B) egocentric and (C) callous/unemotional subscales of the Levenson Self‐Report Psychopathy scale, (D) but not with the antisocial subscale.

Reduced aversion to harming others during the task was significantly associated with total psychopathy ratings (*b* = −0.0083, SE = 0.0028, *t* = −3.00, df = 38, *p = *0.005; Figure 4A), and specifically with egocentric traits (*b* = −0.019, SE = 0.007, *t* = −2.91, df = 38, *p* = 0.006; Figure 4B) and callous/unemotional traits (*b* = −0.042, SE = 0.014, *t* = −3.07, df = 38, *p* = 0.004; Figure 4C) but not antisocial psychopathy traits (*b* = 0.00, *t* = −0.026, *p* = 0.98; Figure 4D). There were no significant associations between aversion to harming oneself and any psychopathic personality traits (total psychopathy: *b* = −0.0063, SE = 0.0032, *t* = −1.94, df = 38, *p* = 0.06; egocentric: *b* = −0.014, SE = 0.008, *t* = −1.75, df = 38, *p* = 0.09; antisocial: *b* = 0.0038, SE = 0.0135, *t* = 0.28, df = 38, *p = *0.78), except callous/unemotional traits (*b* = −0.033, SE = 0.016, *t* = −2.08, df = 38, *p* = 0.04). The inverse correlation between aversion to harming others and callous/unemotional psychopathic personality traits remained significant when restricted to bvFTD (Supplementary Results, Table S19).

### 3.5 Reduced aversion to harming others is associated with impaired perspective taking and empathic concern

**FIGURE 5:**
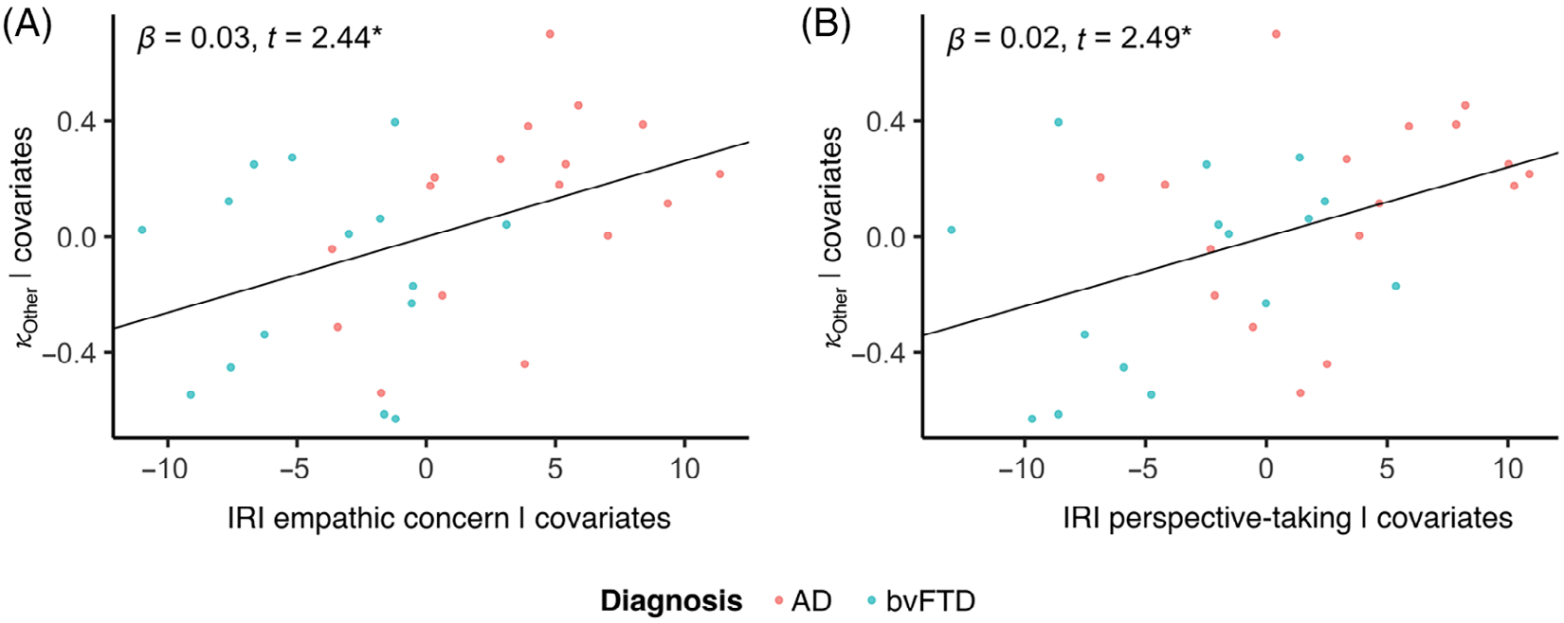
Reduced aversion to harming others is associated with impaired socioemotional function. Reduced aversion to harming others was associated with (A) impaired empathic concern and (B) impaired perspective taking.

There was a significant association between aversion to harming others and empathic concern (*b* = 0.026, SE = 0.011, *t* = 2.44, df = 27, *p* = 0.02; Figure 5A), as well as perspective taking (*b* = 0.024, SE = 0.010, *t* = 2.49, df = 27, *p *= 0.02; Figure 5B), but there were no significant associations between aversion to harming oneself and the same socioemotional measures (empathic concern: *b* = 0.015, SE = 0.013, *t* = 1.18, df = 27, *p* = 0.25; perspective taking: *b* = 0.019, SE = 0.012, *t* = 1.67, df = 27, *p* = 0.11).

### 3.6 Alterations to harm aversion are unrelated to changes in executive function

We examined correlations between harm aversion scores and the ACWMEF construct score to assess relationships with executive functioning. Executive functioning was unrelated to either aversion to harming oneself (*b* = 0.026, SE = 0.033, *t* = 0.776, df = 39, *p* = 0.44) as well as aversion to harming others (*b* = 0.028, SE = 0.032, *t* = 0.862, df = 39, *p* = 0.39).

### 3.7 Reduced aversion to harming others is associated with right vmPFC/OFC atrophy

Reduced aversion to harming others was significantly associated with reduced cortical thickness within the right vmPFC/OFC (*b* = 0.21, SE = 0.09, *t* = 2.31, df = 35, *p* < 0.03). In the left hemisphere, there was a trend toward an association between reduced aversion to harming others and reduced cortical thickness within the left vmPFC/OFC, although the association was not statistically significant (*b* = 0.19, SE = 0.10, *t* = 1.89, df = 35, *p* = 0.07). Aversion to harming oneself was not significantly associated with vmPFC/OFC cortical thickness (left vmPFC/OFC: *b* = 0.025, SE = 0.094, *t* = 0.265, df = 35, *p* = 0.79; right vmPFC/OFC: *b* = 0.044, SE = 0.091, *t* = 0.49, df = 35, *p* = 0.63).

**FIGURE 6:**
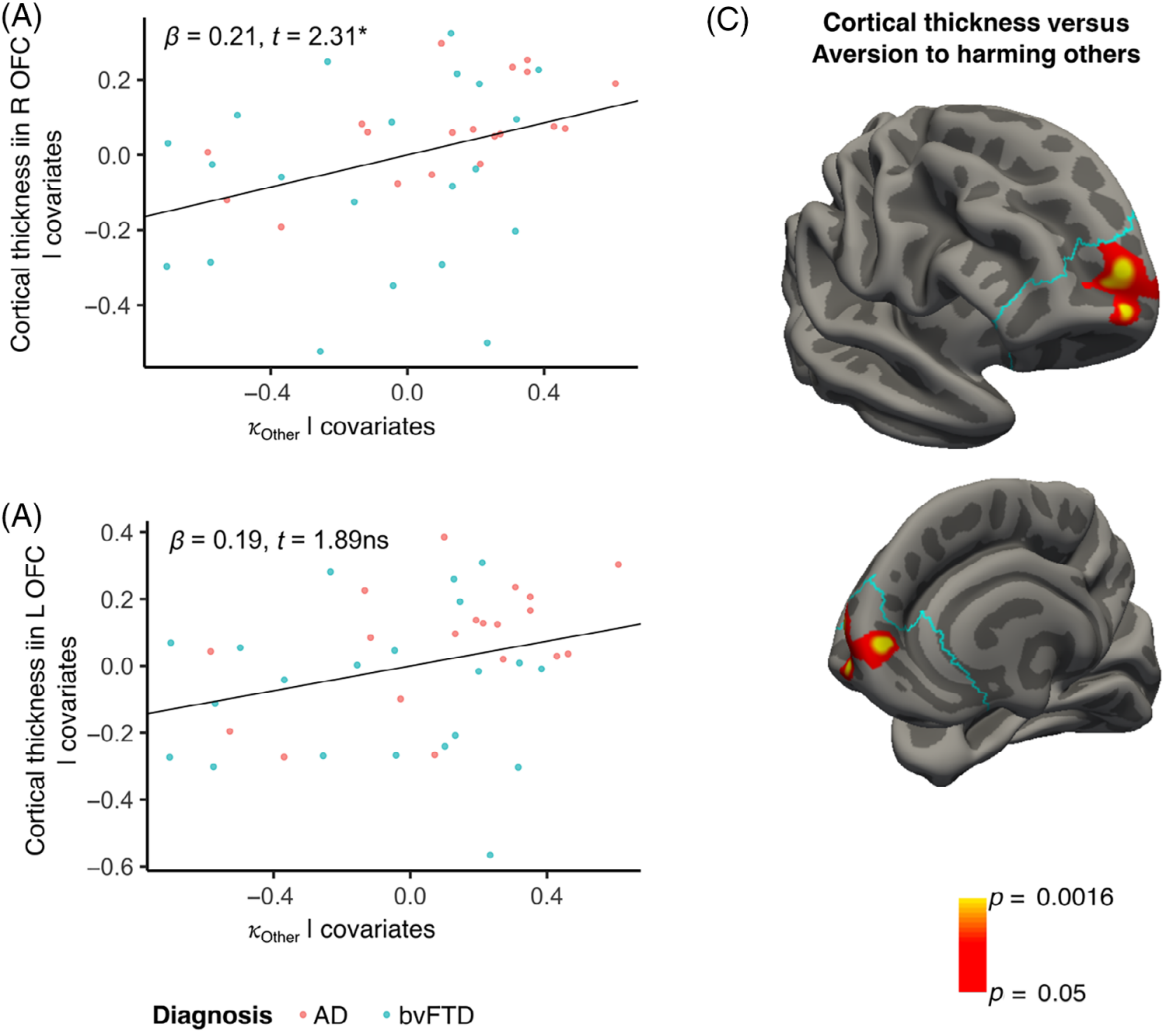
Atrophy in the right vmPFC/OFC is associated with reduced aversion to harming others. Reduced aversion to harming others was (A) associated with reduced cortical thickness in the right vmPFC/OFC but (B) unrelated to cortical thickness in the left vmPFC/OFC. (C) A vertex‐wise model for cortical thickness within the right OFC showed that the association between cortical thickness and aversion to harming others localized primarily to Brodmann area 10. vmPFC, ventromedial prefrontal cortex; OFC, orbitofrontal cortex.

Vertex‐wise analysis masked to the right vmPFC/OFC identified a cluster within the medial frontal pole (Brodmann area 10) that was significantly associated with aversion to harming others (Figure 6). No significant clusters were identified relating vmPFC/OFC cortical thickness with aversion to harming oneself. The supplementary whole‐brain analyses relating vertex‐wise cortical thickness versus aversion to harming oneself or harming others yielded no significant results in other regions.

## 4 DISCUSSION

Using a recently developed moral neuroeconomics decision‐making task, we found that bvFTD patients had reduced harm aversion compared with AD patients. We further found that reduced aversion to harming others was associated with informant‐based measures of non‐aggressive rule‐breaking antisocial behaviors, psychopathic personality traits, impaired empathic concern, and impaired perspective taking in patients with dementia. Finally, we found that reduced aversion to harming others was associated with atrophy within the right vmPFC/OFC, specifically medial frontal pole (Brodmann area 10). Taken together, our results support the hypothesis that reduced aversion to harming others is associated with new onset antisocial behavior and psychopathic personality traits in patients with dementia, related to right vmPFC/OFC atrophy.

### 4.1 Reduced harm aversion in bvFTD using a novel social neuroeconomics task

We found that patients with bvFTD had reduced aversion both to harming other persons and to harming oneself in a moral neuroeconomics task adapted from Crockett et al.^26^, ^27^, ^28^ To our knowledge, this type of task has not previously been used in patients with dementia. It is important to consider whether the alterations in bvFTD reflect a specific insensitivity to harm in others or a more general diminished sensitivity to aversive outcomes. For instance, prior work has found that bvFTD patients have reduced aversion to monetary losses in non‐social neuroeconomics tasks,^18^ reduced aversion to unpleasant smells,^44^ and reduced aversion to negative facial expressions.^45^ We observed significant relationships between task performance and social behavior for aversion to harming others but not aversion to harming oneself, suggesting that reduced sensitivity to negative outcomes in social contexts may be a particularly sensitive and meaningful marker for dysfunction in bvFTD. However, we often observed a trend toward significant relationships between social behavior and aversion to harming oneself, and in supplementary analyses there was no significant difference in the relationships between social behavior and harm aversion to oneself versus others (see Supplementary Materials). Future studies comparing aversion to negative stimuli in social versus non‐social settings tasks will provide further insight into the relative significance of domain‐general versus domain‐specific insensitivity to negative outcomes in bvFTD.

### 4.2 Reduced aversion to harming others during decision‐making tasks is associated with antisocial behaviors and psychopathic personality traits in patients with dementia

Our study found that reduced aversion to harming others on the moral neuroeconomics task was associated with informant ratings for non‐aggressive/rule‐breaking antisocial behavior, egocentric and callous/unemotional psychopathic personality traits, and socioemotional dysfunction, specifically impaired empathic concern and perspective taking. In contrast, task performance was not related to measures of aggressive antisocial behaviors on the SBQ or antisocial behavior on the LSRP, which included items for aggressive behaviors and emotional reactivity such as “has been in a lot of shouting matches with other people” and “when frustrated, I let off steam by blowing my top.”

One interpretation for these findings is that reduced aversion to harming others on the moral neuroeconomics task used in the current study captures the impaired decision‐making processes leading to several (but not all) of the most problematic social behaviors seen in patients with dementia. Specifically, diminished sensitivity to negative outcomes in social contexts leads to reduced care or concern for the suffering of others (IRI‐empathic concern, callous/unemotional traits), reduced consideration for other persons (IRI‐perspective taking, egocentric traits), and reduced consideration for how actions relate to social norms or expectations from society (SBQ‐rule‐breaking behaviors). In contrast, although aggressive behaviors on the SBQ and LSRP were also elevated in bvFTD patients, these behaviors did not relate to impaired aversion to harming others on the moral neuroeconomics task.

These findings are consistent with an extensive body of literature showing that aggressive and non‐aggressive/rule‐breaking behaviors are behaviorally distinct,^46^, ^47^, ^48^ including in bvFTD,^8^ and are associated with different etiologies and developmental trajectories.^46^ Aggressive behaviors manifest earlier in development, are more consistent over time, are more closely linked to genetic factors,^46^, ^49^, ^50^ and relate to autonomic function differences like resting heart rate^51^ and to negative affect and emotional reactivity.^52^, ^53^ In contrast, rule‐breaking behaviors peak in adolescence, are more variable over time, and are more strongly influenced by environmental factors and learning than aggressive behaviors.^46^, ^49^, ^50^

Consistent with these prior distinctions, our results indicate that reduced aversion to harming others may be an important risk factor for patients with dementia violating laws or social norms. Our results raise the hypothesis that reduced aversion to harming others could also lead to rule‐breaking antisocial behavior in other contexts and populations, whether that be due to maladaptive learning during childhood and adolescence or from social cognition impairments that may lead to the inadequate ability to learn about expected negative social outcomes as may occur in antisocial personality disorder.^54^ In contrast, aggressive and violent behaviors may relate to other mechanisms such as increased negative affect and emotional reactivity.^52^, ^53^

### 4.3 Reduced aversion to harming others is associated with right vmPFC/OFC atrophy

We found that reduced aversion to harming others was significantly associated with decreased cortical thickness in the right vmPFC/OFC, and specifically within Brodmann area 10 in the medial frontal pole. This is consistent with an extensive body of prior literature identifying vmPFC/OFC abnormalities in many other patient populations with antisocial behavior, including patients with focal brain lesions^55^, ^56^, ^57^, ^58^, ^59^, ^60^, ^61^ and psychopathy.^62^, ^63^ Functional MRI (fMRI) studies in cognitively unimpaired, healthy subjects have also consistently shown increased BOLD signal in frontopolar regions when making moral decisions^26^ and when making neuroeconomics choices.^64^, ^65^, ^66^, ^67^, ^68^ A right‐hemispheric lateralization for social behavior is also consistent with prior work in bvFTD.^69^ However, although we observed a significant association in the right vmPFC/OFC, there may not be strong laterality in this result, as there was a strong trend in the left vmPFC/OFC as well.

Within the right vmPFC/OFC, aversion to harming others was most specifically linked to reduced cortical thickness in Brodmann area 10. Prior research indicated that the ventromedial part of the frontal pole was highly engaged in tasks involving reward, whereas, moving dorsomedially, there is increasingly greater involvement with social processing/theory of mind,^70^ as well as moral decision‐making in particular.^71^ This may allow this region to serve as a critical processing node at the intersection of economic and social decision‐making.

In a prior task‐based fMRI study using a version of the harm aversion task similar to that used in the current study, BOLD activation in the anterior vmPFC/OFC was associated with the relative subjective value for choices,^26^ but only when shocks were delivered to oneself but not others. Thus, one interpretation for our findings is that right vmPFC/OFC atrophy impairs modulation of subjective value representations based on whether harm occurs to oneself versus another person. Connectivity of the vmPFC/OFC and specifically the frontopolar region to other regions involved in value‐based decision‐making, such as the ventral striatum, support the hypothesis that differences in harm aversion task performance in the current study relate to impairments in modulating subjective value representations based on social context.^43^, ^72^, ^73^, ^74^

### 4.4 Forensic and legal implications

Patients with dementia can be arrested and charged with crimes when antisocial behaviors violate the law.^1^, ^2^, ^4^, ^6^, ^7^, ^8^, ^9^, ^75^, ^76^, ^77^ A complex and unresolved issue is whether these patients meet legal standards for diminished legal responsibility for these actions.^78^, ^79^, ^80^ It could be argued that diminished sensitivity to negative outcomes in situations where patients with dementia break the law meets the legal standard for reduced capacity to appreciate wrongfulness and/or a reduced capacity to conform conduct to the requirements of the law. However, such arguments would depend on the context and specific criminal acts. Further, our findings at the group level may not easily be applied to specific patients because there is not a clearly defined “cutoff” for impaired harm aversion on the task used in the current study.

### 4.5 Limitations

Our sample size was small and limited to a single center and thus requires replication in a larger and more diverse sample. We tested only a limited number of associations between harm aversion and other behavioral and neuropsychological measures; in particular, we lacked executive function measures in our sample, although we did control for overall cognitive dysfunction. Our patient groups were not matched for sex and age, although controlling for these covariates did not change our results. We chose to compare patients with bvFTD with a disease comparison group (AD) without prominent antisocial behaviors rather than cognitively unimpaired subjects to more specifically demonstrate that differences in task performance relate to the behavioral, neuropsychological, and neuroanatomical changes in bvFTD and were not reflective of general cognitive dysfunction. Future studies comparing task performance to healthy subjects, and also to other related dementia populations like semantic dementia, will be informative.

For ethical reasons, given the vulnerability of dementia patients and the risk of undue harm if patients had difficulties with communicating their discomfort due to physical pain, we modified the experiment so that no shocks were actually administered to the subjects or a stranger. This alteration may adversely affect the generalizability of results and comparability to prior studies of harm aversion involving actual shocks/punishment. However, the harm aversion task has previously been administered with hypothetical rather than actual shocks, and primary findings were generally reproduced, including prosocial behavior indicated by greater aversion to harming others than harming oneself.^81^ These findings suggest that our modified task with hypothetical shocks should therefore yield results comparable to those involving actual shocks.

## AUTHOR CONTRIBUTIONS

R. Ryan Darby contributed design or conceptualization of the study, analysis or interpretation of the data, and drafting or revising of the manuscript for intellectual content. Tony X. Phan contributed to acquisition and analysis or interpretation of data and drafting or revising of the manuscript for intellectual content. Jayden J. Lee, Simon Vandekar, Jenifer Z. Siegel, Hongbo Yu, Ciaran M. Considine, and David H. Zald contributed to analysis or interpretation of the data and drafting or revising of the manuscript for intellectual content. Jerica E. Reeder contributed to acquisition and analysis or interpretation of the data.

## CONFLICT OF INTEREST STATEMENT

The authors have no competing interests to declare. Author disclosures are available in the supporting information.

## Supporting information



Supporting Information

Supporting Information

## Data Availability

Data supporting the findings of this study are available from the authors upon reasonable request.
