# Supplementary material for: Reduced harm aversion relates to antisocial behaviors and orbitofrontal atrophy in dementia patients
Source: Alzheimers Dement. 2025 Sep 8;21(9):e70623. doi: 10.1002/alz.70623 (PMC12417317; doi:10.1002/alz.70623)
Supplement: Supplementary file 2 — Supporting Information [file ALZ-21-e70623-s001.docx]

**Supplementary Materials**

# Supplementary Methods

## Supplementary details on patient selection

The study protocol was approved by the Vanderbilt University Medical Center Institutional Review Board, and consent was obtained according to the Declaration of Helsinki. Record review by a behavioral neurologist (RRD) confirmed the diagnosis of bvFTD or Alzheimer’s disease (AD) in each patient according to international research criteria.[1,2] Patients at the MCI stage were included if they had clinical and biomarker evidence consistent with a specific underlying neurodegenerative disease (e.g., abnormal PET scan). Patients were required to have a reliable study informant present for the study visit, defined as someone with contact with the patient at least two times a week and able to report on the patient’s behavior.

*Supplementary details on regions of interest*

We defined a composite vmPFC/OFC ROI, including regions commonly referred to as the orbitofrontal cortex, ventromedial prefrontal cortex, and/or orbitomedial prefrontal cortex, based on prior work implicating this region in value-based decision-making tasks, moral decision-making tasks, and antisocial behavior.[3–5] We included the following areas from the Glasser et al. multimodal parcellation: 10r, 10d, 10v, 10pp, a10p, p10p, 47m, 47s, a47r, 11l, 13l, OFC, pOFC, p32, s32, 25, a24. Given the hypothesized functional differences between different parts of the OFC and to more specifically localize task performance, we additionally performed a vertex-wise analysis masked to our vmPFC/OFC ROI testing for associations between cortical thickness and task performance.

## Assessing whether aversion to harming others and aversion to harming self differ in associations with neuropsychological measures

In order to determine if aversion to harming others and aversion to harming self differ in how they are associated with the neuropsychological/behavioral measures (SBQ total and nonaggressive and aggressive subscale scores; LSRP total and antisocial, egocentric, and callous/unemotional subscale scores; and IRI empathic-concern and perspective-taking subscale scores), we conducted linear mixed-effects models. Harm aversion scores were combined across the self and other trials. For each measure, we computed a linear regression with harm aversion as the response variable; the measure of interest, target (the receiver of the shocks, either “self” or “other”), and the measure x target interaction as predictors of interest; and age, sex, and MoCA score as covariates of non-interest. Subject was included as a random intercept.

## Assessing whether associations between aversion to harming others and neuropsychological measures differ between diagnoses

To assess whether the association between aversion to harming others and neuropsychological/behavioral measures (SBQ total and nonaggressive and aggressive subscale scores; LSRP total and antisocial, egocentric, and callous/unemotional subscale scores; and IRI empathic-concern and perspective-taking subscale scores) differ between AD and bvFTD patients, we computed linear mixed-effects models to assess these interactions. We conducted a linear regression with harm aversion as the response variable. For each neuropsychological measure, we computed a linear regression that included the measure of interest, diagnosis, and the measure x diagnosis interaction as predictors of interest. We further included age, sex, and MoCA score as covariates of non-interest.

*Assessing associations between aversion to harming others or harming self and neuropsychological measures, grouped by diagnoses*

To determine whether aversion to harming others or harming self were related to neuropsychological/behavioral measures within diagnoses, we additionally the main analyses separately for the bvFTD and AD/MCI diagnoses. Linear regression models were conducted with harm aversion as the response variable, each behavioral measure as the predictor of interest and age, sex, and MoCA scores as covariates of non-interest.

## Software details

Statistical analyses were performed using the statistical programming language R version 4.4.1 (<https://www.r-project.org/>) with the data.table, stats, and car packages and plotted using ggplot2.

# Supplementary Results

## Assessing whether aversion to harming others and aversion to harming self differ in associations with neuropsychological measures

There were no significant differences between aversion to harming others and aversion to harming self with regard to the association between aversion and the neuropsychological measures of interest (SBQ total and nonaggressive and aggressive subscale scores; LSRP total and antisocial, egocentric, and callous/unemotional subscale scores; and IRI empathic-concern and perspective-taking subscale scores). Further details of results are presented in Supplementary Tables 1–9.

## Assessing whether associations between aversion to harming others and neuropsychological measures differ between diagnoses

There were no significant differences between AD and bvFTD subjects in relationship between aversion to harming others and the neuropsychological measures of interest (SBQ total and nonaggressive and aggressive subscale scores; LSRP total and antisocial, egocentric, and callous/unemotional subscale scores; and IRI empathic-concern and perspective-taking subscale scores). Further details of results are presented in Supplementary Tables 10–18.

*Assessing associations between harming others or harming self and neuropsychological measures, grouped by diagnoses*

We repeated the analyses of harm aversion and the behavioral variables, grouped by diagnosis, and found that the associations did not remain significant except for the inverse correlation between aversion to harming others and callus/unemotional psychopathic personality traits within bvFTD. Otherwise, the associations were observed across the variance of the entire sample of both diagnostic groups (Supplementary Table 19).

# Supplementary Tables

# Supplementary Table 1. Results for model assessing how aversion to harming others and aversion to harming self differ in association with total SBQ score (antisocial behaviors)^a^

| **Variable** | **b** | **SE** | **df** | **t** | **P** |
| --- | --- | --- | --- | --- | --- |
| Intercept | 0.304 | 0.462 | 38.4 | 0.658 | 0.51 |
| Total SBQ score | -0.00318 | 0.00504 | 52.9 | -0.632 | 0.53 |
| Target (other > self) | 0.0383 | 0.0664 | 41.0 | 0.576 | 0.57 |
| Age | -0.00514 | 0.00582 | 38.0 | -0.882 | 0.38 |
| Female gender | 0.208 | 0.131 | 38.0 | 1.59 | 0.12 |
| MoCA | 0.0273 | 0.0115 | 38.0 | 2.37 | 0.02 |
| Total SBQ score x target (other > self) | 0.00112 | 0.00414 | 41.0 | 0.272 | 0.79 |

^a^ MoCA = Montreal Cognitive Assessment, SBQ = Social Behavior Questionnaire

# Supplementary Table 2. Results for model assessing how aversion to harming others and aversion to harming self differ in association with SBQ nonaggressive subscale score ^a^

| **Variable** | **b** | **SE** | **df** | **T** | **P** |
| --- | --- | --- | --- | --- | --- |
| Intercept | 0.308 | 0.436 | 38.4 | 0.707 | 0.48 |
| SBQ nonaggressive subscale score | -0.0161 | 0.0106 | 54.9 | -1.51 | 0.14 |
| Target (other > self) | 0.0665 | 0.0641 | 41.0 | 1.04 | 0.31 |
| Age | -0.00481 | 0.00550 | 38.0 | -0.874 | 0.39 |
| Female gender | 0.150 | 0.127 | 38.0 | 1.18 | 0.25 |
| MoCA | 0.0285 | 0.0110 | 38.0 | 2.58 | 0.01 |
| SBQ nonaggressive subscale score x TargetOther | -0.00452 | 0.00921 | 41.0 | -0.491 | 0.63 |

^a^ MoCA = Montreal Cognitive Assessment, SBQ = Social Behavior Questionnaire

# Supplementary Table 3. Results for model assessing how aversion to harming others and aversion to harming self differ in association with SBQ aggressive subscale score^a^

| **Variable** | **b** | **SE** | **df** | **t** | **P** |
| --- | --- | --- | --- | --- | --- |
| Intercept | 0.254 | 0.476 | 38.4 | 0.534 | 0.60 |
| SBQ aggressive subscale score | -0.000691 | 0.00662 | 52.6 | -0.104 | 0.92 |
| Target (other > self) | 0.0409 | 0.0665 | 41.0 | 0.615 | 0.54 |
| Age | -0.00459 | 0.00598 | 38.0 | -0.767 | 0.45 |
| Female gender | 0.232 | 0.131 | 38.0 | 1.77 | 0.08 |
| MoCA | 0.0264 | 0.0115 | 38.0 | 2.29 | 0.03 |
| SBQ aggressive subscale score x Target (other > self) | 0.00110 | 0.00537 | 41.0 | 0.205 | 0.84 |

^a^ MoCA = Montreal Cognitive Assessment, SBQ = Social Behavior Questionnaire

# Supplementary Table 4. Results for model assessing how aversion to harming others and aversion to harming self differ in association with IRI empathic-concern subscale score^a^

| **Variable** | **b** | **SE** | **df** | **t** | **P** |
| --- | --- | --- | --- | --- | --- |
| Intercept | -0.614 | 0.574 | 29.4 | -1.07 | 0.29 |
| IRI empathic-concern subscale score | 0.0193 | 0.0116 | 42.3 | 1.66 | 0.10 |
| Target (other > self) | 0.00927 | 0.236 | 30.0 | 0.0393 | 0.97 |
| Age | -0.000193 | 0.00646 | 27.0 | -0.0299 | 0.98 |
| Female gender | 0.188 | 0.129 | 27.0 | 1.46 | 0.16 |
| MoCA | 0.0342 | 0.0121 | 27.0 | 2.83 | 0.009 |
| IRI empathic-concern subscale score x Target (other > self) | 0.00309 | 0.0112 | 30.0 | 0.275 | 0.79 |

^a^ MoCA = Montreal Cognitive Assessment, IRI = Interpersonal Reactivity Index

# Supplementary Table 5. Results for model assessing how aversion to harming others and aversion to harming self differ in association with IRI perspective-taking subscale score^a^

| **Variable** | **b** | **SE** | **df** | **t** | **P** |
| --- | --- | --- | --- | --- | --- |
| Intercept | -0.109 | 0.507 | 28.3 | -0.214 | 0.83 |
| IRI perspective-taking subscale score | 0.0192 | 0.0103 | 43.6 | 1.85 | 0.07 |
| Target (other > self) | 0.00271 | 0.157 | 30.0 | 0.0172 | 0.99 |
| Age | -0.00429 | 0.00663 | 27.0 | -0.648 | 0.52 |
| Female gender | 0.188 | 0.124 | 27.0 | 1.52 | 0.14 |
| MoCA | 0.0287 | 0.0112 | 27.0 | 2.57 | 0.02 |
| IRI perspective-taking subscale score x Target (other > self) | 0.00503 | 0.0103 | 30.0 | 0.487 | 0.63 |

^a^ MoCA = Montreal Cognitive Assessment, IRI = Interpersonal Reactivity Index

# Supplementary Table 6. Results for model assessing how aversion to harming others and aversion to harming self differ in association with total LSRP score^a^

| **Variable** | **b** | **SE** | **df** | **t** | **P** |
| --- | --- | --- | --- | --- | --- |
| Intercept | 0.784 | 0.470 | 40.2 | 1.67 | 0.10 |
| Total LSRP score | -0.00665 | 0.00293 | 55.1 | -2.26 | 0.03 |
| Target (other > self) | 0.124 | 0.159 | 41.0 | 0.779 | 0.44 |
| Age | -0.00828 | 0.00543 | 38.0 | -1.52 | 0.14 |
| Female gender | 0.144 | 0.118 | 38.0 | 1.21 | 0.23 |
| MoCA | 0.0324 | 0.0107 | 38.0 | 3.03 | 0.004 |
| Total LSRP score x Target (other > self) | -0.00128 | 0.00256 | 41.0 | -0.499 | 0.62 |

^a^ MoCA = Montreal Cognitive Assessment, LSRP = Levenson Self-Report Psychopathy scale

# Supplementary Table 7. Results for model assessing how aversion to harming others and aversion to harming self differ in association with LSRP antisocial subscale score^a^

| **Variable** | **b** | **SE** | **df** | **t** | **P** |
| --- | --- | --- | --- | --- | --- |
| Intercept | 0.156 | 0.551 | 39.4 | 0.284 | 0.78 |
| LSRP antisocial subscale score | 0.00522 | 0.0127 | 53.3 | 0.411 | 0.68 |
| Target (other > self) | 0.139 | 0.146 | 41.0 | 0.954 | 0.35 |
| Age | -0.00423 | 0.00616 | 38.0 | -0.686 | 0.50 |
| Female gender | 0.236 | 0.126 | 38.0 | 1.87 | 0.07 |
| MoCA | 0.0265 | 0.0115 | 38.0 | 2.30 | 0.03 |
| LSRP antisocial subscale score x Target (other > self) | -0.00699 | 0.0105 | 41.0 | -0.664 | 0.51 |

# Supplementary Table 8. Results for model assessing how aversion to harming others and aversion to harming self differ in association with LSRP callous subscale score^a^

| **Variable** | **b** | **SE** | **df** | **t** | **P** |
| --- | --- | --- | --- | --- | --- |
| Intercept | 0.481 | 0.423 | 39.4 | 1.14 | 0.26 |
| LSRP callous subscale score | -0.0367 | 0.0146 | 55.6 | -2.52 | 0.01 |
| Target (other > self) | 0.0652 | 0.116 | 41.0 | 0.563 | 0.58 |
| Age | -0.00647 | 0.00526 | 38.0 | -1.23 | 0.23 |
| Female gender | 0.158 | 0.116 | 38.0 | 1.37 | 0.18 |
| MoCA | 0.0367 | 0.0110 | 38.0 | 3.34 | 0.002 |
| LSRP callous subscale score x Target (other > self) | -0.00203 | 0.0129 | 41.0 | -0.157 | 0.88 |

^a^ MoCA = Montreal Cognitive Assessment, LSRP = Levenson Self-Report Psychopathy scale

# Supplementary Table 9. Results for model assessing how aversion to harming others and aversion to harming self differ in association with LSRP egocentric subscale score^a^

| **Variable** | **b** | **SE** | **df** | **t** | **P** |
| --- | --- | --- | --- | --- | --- |
| Intercept | 0.714 | 0.466 | 39.4 | 1.53 | 0.13 |
| LSRP egocentric subscale score | -0.0146 | 0.00703 | 53.7 | -2.08 | 0.04 |
| Target (other > self) | 0.121 | 0.126 | 41.0 | 0.959 | 0.34 |
| Age | -0.00776 | 0.00545 | 38.0 | -1.42 | 0.16 |
| Female gender | 0.101 | 0.126 | 38.0 | 0.804 | 0.43 |
| MoCA | 0.0296 | 0.0107 | 38.0 | 2.77 | 0.009 |
| LSRP egocentric subscale score x Target (other > self) | -0.00371 | 0.00590 | 41.0 | -0.628 | 0.53 |

^a^ MoCA = Montreal Cognitive Assessment, LSRP = Levenson Self-Report Psychopathy scale

# Supplementary Table 10. Results for model assessing whether association between aversion to harming others and total SBQ score differs between AD and bvFTD^a^

| **Variable** | **b** | **SE** | **t** | **P** |
| --- | --- | --- | --- | --- |
| Intercept | 0.382 | 0.531 | 0.719 | 0.48 |
| Total SBQ score | 0.00729 | 0.0117 | 0.625 | 0.54 |
| bvFTD diagnosis | -0.322 | 0.165 | -1.96 | 0.06 |
| Age | -0.00608 | 0.00639 | -0.951 | 0.35 |
| Female gender | 0.103 | 0.142 | 0.725 | 0.47 |
| MoCA | 0.0365 | 0.0117 | 3.13 | 0.003 |
| Total SBQ score x bvFTD diagnosis | -0.00573 | 0.0129 | -0.443 | 0.66 |

^a^ bvFTD = behavioral variant frontotemporal dementia, MoCA = Montreal Cognitive Assessment, SBQ = Social Behavior Questionnaire

# Supplementary Table 11. Results for model assessing whether association between aversion to harming others and SBQ nonaggressive subscale score differs between AD and bvFTD^a^

| **Variable** | **b** | **SE** | **t** | **P** |
| --- | --- | --- | --- | --- |
| Intercept | 0.381 | 0.531 | 0.718 | 0.48 |
| SBQ nonaggressive subscale score | -0.0132 | 0.0668 | -0.198 | 0.84 |
| bvFTD diagnosis | -0.250 | 0.161 | -1.55 | 0.13 |
| Age | -0.00567 | 0.00637 | -0.890 | 0.38 |
| Female gender | 0.0512 | 0.142 | 0.361 | 0.72 |
| MoCA | 0.0375 | 0.0116 | 3.25 | 0.003 |
| SBQ nonaggressive subscale score x bvFTD diagnosis | 0.000300 | 0.0680 | 0.00441 | > .99 |

^a^ MoCA = Montreal Cognitive Assessment, SBQ = Social Behavior Questionnaire

# Supplementary Table 12. Results for model assessing whether association between aversion to harming others and SBQ aggressive subscale score differs between AD and bvFTD^a^

| **Variable** | **b** | **SE** | **t** | **P** |
| --- | --- | --- | --- | --- |
| Intercept | 0.382 | 0.522 | 0.733 | 0.47 |
| SBQ aggressive subscale score | 0.00677 | 0.0123 | 0.551 | 0.59 |
| bvFTD diagnosis | -0.371 | 0.161 | -2.31 | 0.03 |
| Age | -0.00589 | 0.00633 | -0.932 | 0.36 |
| Female gender | 0.102 | 0.141 | 0.725 | 0.47 |
| MoCA | 0.0360 | 0.0115 | 3.12 | 0.004 |
| SBQ aggressive subscale score x bvFTD diagnosis | -0.000642 | 0.0142 | -0.0453 | 0.96 |

^a^ bvFTD = behavioral variant frontotemporal dementia, MoCA = Montreal Cognitive Assessment, SBQ = Social Behavior Questionnaire

# Supplementary Table 13. Results for model assessing whether association between aversion to harming others and IRI empathic-concern subscale score differs between AD and bvFTD^a^

| **Variable** | **b** | **SE** | **t** | **P** |
| --- | --- | --- | --- | --- |
| Intercept | -1.39 | 1.04 | -1.34 | 0.19 |
| IRI empathic-concern subscale score | 0.0437 | 0.0311 | 1.40 | 0.17 |
| bvFTD diagnosis | 0.686 | 0.862 | 0.796 | 0.43 |
| Age | 0.00296 | 0.00767 | 0.386 | 0.70 |
| Female gender | 0.112 | 0.145 | 0.776 | 0.44 |
| MoCA | 0.0400 | 0.0147 | 2.72 | 0.01 |
| IRI empathic-concern subscale score | -0.0377 | 0.0394 | -0.958 | 0.35 |

^a^ bvFTD = behavioral variant frontotemporal dementia, MoCA = Montreal Cognitive Assessment, IRI = Interpersonal Reactivity Index

# Supplementary Table 14. Results for model assessing whether association between aversion to harming others and IRI perspective-taking subscale score differs between AD and bvFTD^a^

| **Variable** | **b** | **SE** | **t** | **P** |
| --- | --- | --- | --- | --- |
| Intercept | -0.382 | 0.632 | -0.604 | 0.55 |
| IRI perspective-taking subscale score | 0.0143 | 0.0179 | 0.802 | 0.43 |
| bvFTD diagnosis | -0.232 | 0.388 | -0.598 | 0.56 |
| Age | -0.000231 | 0.00744 | -0.0311 | 0.98 |
| Female gender | 0.139 | 0.142 | 0.976 | 0.34 |
| MoCA | 0.0411 | 0.0127 | 3.23 | 0.003 |
| IRI perspective-taking subscale score x bvFTD diagnosis | 0.00609 | 0.0263 | 0.231 | 0.82 |

^a^ bvFTD = behavioral variant frontotemporal dementia, MoCA = Montreal Cognitive Assessment, IRI = Interpersonal Reactivity Index

# Supplementary Table 15. Results for model assessing whether association between aversion to harming others and total LSRP scale differs between AD and bvFTD^a^

| **Variable** | **b** | **SE** | **t** | **P** |
| --- | --- | --- | --- | --- |
| Intercept | 0.442 | 0.717 | 0.616 | 0.54 |
| Total LSRP scale | -0.00250 | 0.00875 | -0.286 | 0.78 |
| bvFTD diagnosis | 0.0491 | 0.507 | 0.0968 | 0.92 |
| Age | -0.00634 | 0.00623 | -1.02 | 0.32 |
| Female gender | 0.0673 | 0.132 | 0.510 | 0.61 |
| MoCA | 0.0417 | 0.0117 | 3.56 | 0.001 |
| Total LSRP scale x bvFTD diagnosis | -0.00438 | 0.00987 | -0.444 | 0.66 |

^a^ bvFTD = behavioral variant frontotemporal dementia, MoCA = Montreal Cognitive Assessment, LSRP = Levenson Self-Report Psychopathy scale

# Supplementary Table 16. Results for model assessing whether association between aversion to harming others and LSRP antisocial subscale score differs between AD and bvFTD^a^

| **Variable** | **b** | **SE** | **t** | **P** |
| --- | --- | --- | --- | --- |
| Intercept | 0.262 | 0.627 | 0.419 | 0.68 |
| LSRP antisocial subscale score | 0.00964 | 0.0172 | 0.562 | 0.58 |
| bvFTD diagnosis | -0.351 | 0.354 | -0.990 | 0.33 |
| Age | -0.00561 | 0.00656 | -0.856 | 0.40 |
| Female gender | 0.0783 | 0.137 | 0.573 | 0.57 |
| MoCA | 0.0372 | 0.0117 | 3.17 | 0.003 |
| LSRP antisocial subscale score x bvFTD diagnosis | -0.000846 | 0.0245 | -0.0345 | 0.97 |

^a^ bvFTD = behavioral variant frontotemporal dementia, MoCA = Montreal Cognitive Assessment, LSRP = Levenson Self-Report Psychopathy scale

# Supplementary Table 17. Results for model assessing whether association between aversion to harming others and LSRP callous subscale score differs between AD and bvFTD^a^

| **Variable** | **b** | **SE** | **t** | **P** |
| --- | --- | --- | --- | --- |
| Intercept | 0.107 | 0.491 | 0.217 | 0.83 |
| LSRP callous subscale score | 0.0443 | 0.0456 | 0.972 | 0.34 |
| bvFTD diagnosis | 0.320 | 0.307 | 1.04 | 0.30 |
| Age | -0.00765 | 0.00590 | -1.30 | 0.20 |
| Female gender | 0.0352 | 0.129 | 0.272 | 0.79 |
| MoCA | 0.0476 | 0.0115 | 4.12 | < .001 |
| LSRP callous subscale score x bvFTD diagnosis | -0.0889 | 0.0496 | -1.79 | 0.08 |

^a^ bvFTD = behavioral variant frontotemporal dementia, MoCA = Montreal Cognitive Assessment, LSRP = Levenson Self-Report Psychopathy scale

# Supplementary Table 18. Results for model assessing whether association between aversion to harming others and LSRP egocentric subscale score differs between AD and bvFTD^a^

| **Variable** | **b** | **SE** | **t** | **P** |
| --- | --- | --- | --- | --- |
| Intercept | 0.625 | 0.710 | 0.881 | 0.38 |
| LSRP egocentric subscale score | -0.0113 | 0.0271 | -0.417 | 0.68 |
| bvFTD diagnosis | -0.156 | 0.436 | -0.357 | 0.72 |
| Age | -0.00723 | 0.00610 | -1.19 | 0.24 |
| Female gender | 0.0202 | 0.136 | 0.149 | 0.88 |
| MoCA | 0.0388 | 0.0116 | 3.33 | 0.002 |
| LSRP egocentric subscale score x bvFTD diagnosis | -0.00307 | 0.0284 | -0.108 | 0.91 |

^a^ bvFTD = behavioral variant frontotemporal dementia, MoCA = Montreal Cognitive Assessment, LSRP = Levenson Self-Report Psychopathy scale

**Supplementary Table 19. Results of models assessing association between aversion to harming others (κ_other_) or harming oneself (κ_self)_**

| **Diagnosis** | **Response** | **Predictor** | **b** | **SE** | **t** | **P** |
| --- | --- | --- | --- | --- | --- | --- |
| bvFTD | κ_other_ | Total SBQ score | 0.00401 | 0.00591 | 0.678 | 0.51 |
| AD | κ_other_ | Total SBQ score | 0.0103 | 0.0118 | 0.867 | 0.40 |
| bvFTD | κ_other_ | SBQ nonaggressive subscale score | -0.00638 | 0.0128 | -0.498 | 0.62 |
| AD | κ_other_ | SBQ nonaggressive subscale score | -0.0150 | 0.0707 | -0.213 | 0.83 |
| bvFTD | κ_other_ | SBQ aggressive subscale score | 0.00744 | 0.00761 | 0.978 | 0.34 |
| AD | κ_other_ | SBQ aggressive subscale score | 0.00932 | 0.0125 | 0.743 | 0.47 |
| bvFTD | κ_other_ | Total LSPR score | -0.00597 | 0.00443 | -1.35 | 0.19 |
| AD | κ_other_ | Total LSPR score | 0.000104 | 0.00924 | 0.0113 | > .99 |
| bvFTD | κ_other_ | LSPR antisocial subscale score | 0.00741 | 0.0180 | 0.412 | 0.69 |
| AD | κ_other_ | LSPR antisocial subscale score | 0.0236 | 0.0182 | 1.30 | 0.21 |
| bvFTD | κ_other_ | LSPR callous subscale score | -0.0477 | 0.0216 | -2.21 | 0.04 |
| AD | κ_other_ | LSPR callous subscale score | 0.0398 | 0.0488 | 0.815 | 0.43 |
| bvFTD | κ_other_ | LSRP egocentric subscale score | -0.0116 | 0.00949 | -1.22 | 0.24 |
| AD | κ_other_ | LSRP egocentric subscale score | -0.00989 | 0.0312 | -0.317 | 0.76 |
| bvFTD | κ_other_ | IRI perspective-taking score | 0.0200 | 0.0197 | 1.02 | 0.33 |
| AD | κ_other_ | IRI perspective-taking score | 0.0126 | 0.0190 | 0.664 | 0.52 |
| bvFTD | κ_other_ | IRI empathic-concern subscale score | -0.0157 | 0.0313 | -0.501 | 0.63 |
| AD | κ_other_ | IRI empathic-concern subscale score | 0.0526 | 0.0289 | 1.82 | 0.09 |
| bvFTD | κ_self_ | Total SBQ score | 0.00251 | 0.00572 | 0.439 | 0.67 |
| AD | κ_self_ | Total SBQ score | 0.0100 | 0.0156 | 0.644 | 0.53 |
| bvFTD | κ_self_ | SBQ nonaggressive subscale score | 0.00247 | 0.0124 | 0.199 | 0.84 |
| AD | κ_self_ | SBQ nonaggressive subscale score | 0.0976 | 0.0886 | 1.10 | 0.29 |
| bvFTD | κ_self_ | SBQ aggressive subscale score | 0.00433 | 0.00743 | 0.583 | 0.57 |
| AD | κ_self_ | SBQ aggressive subscale score | 0.00920 | 0.0164 | 0.560 | 0.58 |
| bvFTD | κ_self_ | Total LSPR score | -0.00138 | 0.00446 | -0.310 | 0.76 |
| AD | κ_self_ | Total LSPR score | -0.00240 | 0.0120 | -0.200 | 0.84 |
| bvFTD | κ_self_ | LSPR antisocial subscale score | 0.0167 | 0.0169 | 0.989 | 0.34 |
| AD | κ_self_ | LSPR antisocial subscale score | 0.0204 | 0.0243 | 0.840 | 0.41 |
| bvFTD | κ_self_ | LSPR callous subscale score | -0.0203 | 0.0229 | -0.888 | 0.39 |
| AD | κ_self_ | LSPR callous subscale score | -0.0126 | 0.0648 | -0.195 | 0.85 |
| bvFTD | κ_self_ | LSRP egocentric subscale score | -0.000851 | 0.00949 | -0.0897 | 0.93 |
| AD | κ_self_ | LSRP egocentric subscale score | -0.0243 | 0.0402 | -0.603 | 0.56 |
| bvFTD | κ_self_ | IRI perspective-taking score | -0.00929 | 0.0185 | -0.501 | 0.63 |
| AD | κ_self_ | IRI perspective-taking score | 0.0317 | 0.0240 | 1.32 | 0.21 |
| bvFTD | κ_self_ | IRI empathic-concern subscale score | -0.0216 | 0.0279 | -0.774 | 0.46 |
| AD | κ_self_ | IRI empathic-concern subscale score | -0.00490 | 0.0434 | -0.113 | 0.91 |

^a^ bvFTD = behavioral variant frontotemporal dementia, SBQ = Social Behavior Questionnaire, MoCA = Montreal Cognitive Assessment, LSRP = Levenson Self-Report Psychopathy scale

# References

[1] Rascovsky K, Hodges JR, Knopman D, Mendez MF, Kramer JH, Neuhaus J, et al. Sensitivity of revised diagnostic criteria for the behavioural variant of frontotemporal dementia. Brain : A Journal of Neurology 2011;134:2456–77. https://doi.org/10.1093/brain/awr179.

[2] McKhann GM, Knopman DS, Chertkow H, Hyman BT, Jack CR, Kawas CH, et al. The diagnosis of dementia due to Alzheimer’s disease: recommendations from the National Institute on Aging-Alzheimer’s Association workgroups on diagnostic guidelines for Alzheimer’s disease. Alzheimer’s & Dementia : The Journal of the Alzheimer’s Association 2011;7:263–9. https://doi.org/10.1016/j.jalz.2011.03.005.

[3] Fumagalli M, Priori A. Functional and clinical neuroanatomy of morality. Brain 2012;135:2006–21. https://doi.org/10.1093/brain/awr334.

[4] FitzGerald THB, Seymour B, Dolan RJ. The role of human orbitofrontal cortex in value comparison for incommensurable objects. J Neurosci 2009;29:8388–95. https://doi.org/10.1523/JNEUROSCI.0717-09.2009.

[5] Séguin JR. Neurocognitive elements of antisocial behavior: Relevance of an orbitofrontal cortex account. Brain Cogn 2004;55:185–97. https://doi.org/10.1016/S0278-2626(03)00273-2.
